# Salt-Sensitive Hypertension in GR^+/−^ Rats Is Accompanied with Dysregulation in Adrenal Soluble Epoxide Hydrolase and Polyunsaturated Fatty Acid Pathways

**DOI:** 10.3390/ijms222413218

**Published:** 2021-12-08

**Authors:** Paul-Emmanuel Vanderriele, Qing Wang, Anne-Marie Mérillat, Frédérique Ino, Gilles Aeschlimann, Xavier Ehret, David Ancin Del Olmo, Verónica Ponce de León, Ute I. Scholl, Denise V. Winter, Alex Odermatt, Edith Hummler, Sophia N. Verouti

**Affiliations:** 1 Department of Biomedical Science, University of Lausanne, 1015 Lausanne, Switzerland; paul-emmanuel.vanderriele@unil.ch (P.-E.V.); anne-marie.merillat@unil.ch (A.-M.M.); frederique.ino@unil.ch (F.I.); gilles1521@hotmail.com (G.A.); xaviergfg@gmail.com (X.E.); davidancin@gmail.com (D.A.D.O.); veronicapdl@gmail.com (V.P.d.L.); Edith.Hummler@unil.ch (E.H.); 2 National Center of Competence in Research Kidney.CH, 8057 Zürich, Switzerland; winter@unibas.ch (D.V.W.); alex.odermatt@unibas.ch (A.O.); 3 Division of Nephrology and Hypertension, Lausanne University Hospital (CHUV), 1015 Lausanne, Switzerland; Qing.Wang@chuv.ch; 4 Department of Nephrology, School of Medicine, Heinrich-Heine-Universität Düsseldorf, 40225 Düsseldorf, Germany; ute.scholl@med.uni-duesseldorf.de; 5 Center of Functional Genomics, Berlin Institute of Health at Charité, Universitätsmedizin Berlin, 10117 Berlin, Germany; 6 Division of Molecular and Systems Toxicology, Department of Pharmaceutical Sciences, University of Basel, 4056 Basel, Switzerland

**Keywords:** adrenal gland hyperplasia, hypertension, glucocorticoid receptor, soluble-epoxide hydrolase, Chrousos syndrome, glucocorticoid resistance

## Abstract

Mutations within the glucocorticoid receptor (GR) gene locus lead to glucocorticoid resistance which is characterized by several clinical symptoms such as adrenal gland hyperplasia and salt-sensitive hypertension, although the underlying mechanisms are still unknown. We studied GR haploinsufficient (GR^+/−^) Sprague Dawley rats which, on a standard diet, showed significantly increased plasma aldosterone and corticosterone levels and an adrenocortex hyperplasia accompanied by a normal systolic blood pressure. Following a high salt diet, these rats developed salt-sensitive hypertension and maintained elevated enzyme-soluble epoxide hydrolase (sEH) in adrenal glands, while sEH was significantly decreased in wild-type rats. Furthermore, GR^+/−^ rats showed dysregulation of the equilibrated linoleic and arachidonic acid pathways, with a significant increase of less active metabolites such as 8,9-DiHETrE. In Sprague Dawley rats, GR haploinsufficiency induced steroid disturbances, which provoked hypertension only in combination with high salt intake, which was accompanied by disturbances in sEH and fatty acid metabolism. Our results suggest that sEH inhibition could be a potential target to treat hypertension in patients with GR haploinsufficiency.

## 1. Introduction

A systematic analysis of population-based studies from 90 countries showed that one-third of the human adult population is hypertensive [1]. It is well established that hypertension and the associated cardiovascular symptoms are one of the leading causes of death worldwide [2].

Glucocorticoids are important for various pathophysiological and physiological processes such as metabolic homeostasis and regulation of blood pressure [3], energy intake, glucose and lipid homeostasis [4]. Glucocorticoid excess can cause hypertension, obesity, insulin resistance/diabetes and Cushing’s syndrome [5]. Previous studies [6,7] have reported excess glucocorticoid secretion in patients with primary aldosteronism that may contribute to associated metabolic risk and diastolic blood pressure [8]. Therefore, it was proposed that treatment with mineralocorticoid receptor antagonists alone may not be sufficient to counteract adverse metabolic risk in patients with primary aldosteronism [8,9].

Glucocorticoid actions are mainly mediated by the glucocorticoid receptor (GR), a nuclear receptor encoded by the *NR3C1* gene [9]. GR mutations result in familial or sporadic glucocorticoid resistance syndrome (PGGR), characterized by a decreased negative feedback of cortisol on the hypothalamic–pituitary–adrenal (HPA) axis, increased secretion of adrenocorticotrophic hormone (ACTH) and cortisol, resistance to adrenal suppression by dexamethasone, and the absence of Cushingoid features [10,11]. The increased adrenal activity also results in increased amounts of adrenal androgens and of mineralocorticoids. In humans, several clinical observations demonstrated that GR mutations might be a cause of hypertension (for a review, see [12]). It is believed that mineralocorticoids together with cortisol are responsible for causing hypertension and/or hypokalemic alkalosis, whereas androgens cause acne, hirsutism, menstrual irregularities, and infertility in women [13]. To date, 35 germinal mutations of the human GR have been identified; most of them are heterozygous missense mutations causing partial loss of function of the GR [14,15,16,17] or nonsense mutations causing GR haploinsufficiency [12,18].

The effects of loss-of-function or decreased GR protein abundance was investigated in three genetically engineered knockout mouse models where exon 2 is targeted (GR^Hypo^ [19]), exon 3 is deleted (GR^Null^ [20]) or a βgeo reporter cassette is integrated in the GR gene (GR^βgeo^ [21]). These mouse models developed a glucocorticoid resistance syndrome. Blood pressure was investigated in GR^βgeo/+^ mice, revealing salt-sensitive hypertension and renal tubular and vascular abnormalities [21,22].

Hypertension is a global health challenge and, recently, soluble epoxide hydrolase (sEH) emerged as a promising target for anti-hypertensive therapies [23]. SEH, encoded by the *ephx2* gene, is an enzyme that regulates the levels of epoxyeicosatrienoic acids (EETs), epoxyeicosatetraenoic acids (EEQs), and epoxydocosapentanoic acid (EDP) by converting them into their inactive diols. Omega-3 and omega-6 polyunsaturated fatty acid metabolites are mainly produced in the endothelium and are known to have an effect in the regulation and vascular tone of pulmonary, renal, and cardiac function [24]. The vasodilatory effect caused by epoxyeicosatrienoic acids (EETs) becomes less potent when metabolised by sEH [25,26]. The higher expression of sEH in Sprague Dawley hypertensive rats highlighted the importance of this enzyme in regulation of cardiovascular function [27,28].

The role of GR haploinsufficiency in hypertension and fatty acid metabolism is not well studied. We therefore generated a GR knockout rat model and assessed adrenal gland structure, steroid production, blood pressure and the amount of fatty acid metabolites in a standard or high salt diet.

## 2. Results

### 2.1. GR^+/−^ Rats Develop Hormonal Disturbances and Adrenal Gland Hyperplasia

Previously [29], using TALEN technology (Transcription Activator-Like Effector Nucleases), we generated a rat model carrying a deletion in the dimerization and/or DNA-binding domain of the GR named GR^em2^. Genotyping (Figure S1A) showed that all the homozygous offspring died perinatally. We isolated and sequenced DNA of the homozygous mutant embryos and identified a 7 bp deletion in exon 3 (Figure S1B). This genomic modification caused the deletion of leucine and cysteine in positions 475 and 476 and shifted the reading frame of the protein, leading to the generation of an early stop codon at position 504 (Figure S1C). GR transcript analysis for exons 2–3 (Figures S1D and S2), exons 2–4 (Figures S1E and S2) or exon 8 (Figures S1F and S2) did not reveal any fragment in homozygous mutant rats, thus demonstrating that the rat em2 mutant allele is a null allele. This was confirmed in protein lysates isolated from rat embryonic fibroblasts (REF) using antibodies directed towards the N- and C- terminal end of GR. Full-length GR protein was detected at 94 kDa in rat embryonic fibroblasts (REF) from wild-type rats (Figure S3A). This protein band was clearly absent in REF extracts from GR^−/−^ embryos, and furthermore, no other fragment was detected (Figure S3A). Additionally, GR expression was analysed in liver and kidney from GR^+/+^ and GR^+/−^ rats, where we detected a 50% and 70% reduction of protein levels, respectively (Figure S3B).

In conclusion, the GR^em2/em2^ rat is a knockout model generated by a mutation shifting the reading frame of the protein and generating a stop (TGA) codon at position 504 in the second zinc finger of the DNA-binding domain of the receptor. In addition, qRT-PCR and Western blot analysis failed to detect any mutated GR fragment suggesting a nonsense-mediated mRNA decay mechanism.

**Figure 1:**
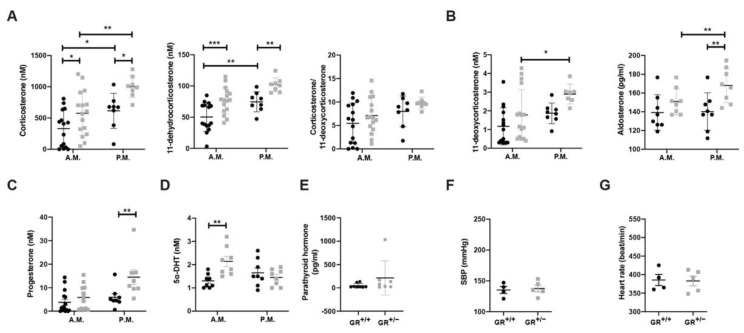
GR^+/−^ rats developed steroid disturbances. Plasma concentrations of (**A**) glucocorticoids, including corticosterone, 11-dehydrocorticosterone, and the corticosterone/11-dehydrocorticosterone ratio, (**B**) mineralocorticoids, including 11-deoxycorticosterone and aldosterone, (**C**) progesterone and (**D**) 5α-DHT during morning (7–8 a.m.) and evening (6–7 p.m.) from 3–4 weeks old male GR^+/+^ (n = 15 − 16) or GR^+/−^ (n = 8) rats. (**E**) Plasma concentration of parathyroid hormone from 3–4-week-old male GR^+/+^ (n = 15 − 16) or GR^+/−^ (n = 8) rats. (**F**) Systolic blood pressure and (**G**) heart rate of 3–4-week-old male GR^+/+^ (n = 4) or GR^+/−^ (n = 5) rats in standard diet (StD). Values are indicated as mean ± SEM, and data were evaluated by two-way ANOVA and compared with Fisher’s LSD test or unpaired two-tailed t test, and differences were assessed at * *p* < 0.05; ** *p* < 0.01; *** *p* < 0.001.

GR haploinsufficiency results in familial or sporadic glucocorticoid resistance syndrome characterized by increased secretion of cortisol. In order to identify any hormonal disturbances in our animal model, we checked the plasma levels of the main hormones in the morning (7–8 a.m.) and afternoon (6–7 p.m.) in GR^+/+^ and GR^+/−^ rats (Figure 1). Glucocorticoid levels were measured in plasma, and both corticosterone and 11-dehydrocorticosterone were elevated in GR^+/−^ rats compared to GR^+/+^ rats in the morning and afternoon (Figure 1A). 11β-HSD2 activity estimated by the corticosterone to 11-dehydrocorticosterone ratio was not different between GR^+/+^ and GR^+/−^ rats (Figure 1A). Plasma levels of the mineralocorticoid 11-deoxycorticosterone (Figure 1B, left panel) were similar between GR^+/+^ and GR^+/−^ rats, whereas the levels of aldosterone, the main mineralocorticoid hormone produced by zona glomerulosa, was higher in GR^+/−^ rats in the afternoon (Figure 1B, right panel). Progesterone (Figure 1C) was higher in the afternoon and 5α-DHT levels were higher in the morning (Figure 1D), while disturbances were also observed in other androgens (Table S3). Parathyroid hormone (measured only in the morning) was similar between wild-type and GR^+/−^ rats (Figure 1E).

GR haploinsufficiency in combination with steroid disturbances is indicative for a glucocorticoid resistance syndrome which may lead to hypertension. However, under standard salt diet, both wild-type and GR^+/−^ rats had similar mean blood pressure (Figure 1F) and heart rate (Figure 1G).

**Figure 2:**
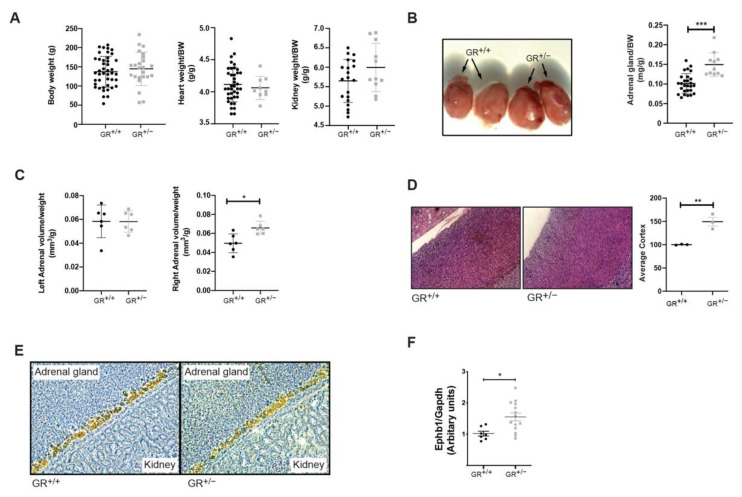
GR^+/−^ rats have enlarged adrenal glands (**A**) Body weight (GR^+/+^ n = 24; GR^+/−^ n = 43), heart weight (GR^+/+^ n = 9; GR^+/−^ n = 39) and kidney weight (GR^+/+^ n = 11; GR^+/−^ n = 20) per body weight. (**B**) Pictures and weight of adrenal glands per body weight from 3-weeks old GR^+/+^ (n = 25) and GR^+/−^ (n = 11) male rats. (**C**) Volume per body weight of adrenal glands from 8-weeks old GR^+/+^ (n = 12) and GR^+/−^ (n = 11) male rats. (**D**) H&E staining and its quantification of the size of the *zona fasciculata* of adrenal glands from 3-week-old GR^+/+^ (n = 3) and GR^+/−^ (n = 3) male rats. (**E**) CYP11B2 staining of the zona glomerulosa of adrenal glands from 3-week-old GR^+/+^ (n = 3) and GR^+/−^ male rats (n = 3). (**F**) Gene expression of *Ephb1* in the adrenal glands of GR^+/+^ (n = 7) and GR^+/−^ rats (n = 14) in StD. Values are indicated as mean ± SEM, and data were evaluated by unpaired two-tailed *t* test and differences assessed at * *p* < 0.05, ** *p* < 0.01 or *** *p* < 0.001.

Under standard salt diet, despite similar body weight, heart and kidney weight of GR^+/+^ and GR^+/−^ rats (Figure 2A), the adrenal gland size was significantly increased in GR^+/−^ rats (about 30%) (Figure 2B), and echography revealed unilateral adrenal gland hyperplasia (right adrenal gland) and a tendency to a higher adrenal volume in GR^+/−^ rats (Figure 2C). Histological analysis revealed that adrenal cortex was enlarged in GR^+/−^ compared to GR^+/+^ rats (Figure 2D) and cyp11B2 staining indicated that zona glomerulosa did not differ between GR^+/+^ and GR^+/−^ rats (Figure 2E). Measurements of adrenal cell size in the different zones (glomerulosa, fasciculata and reticularis) did not differ between genotypes (data not shown), suggesting that the larger adrenal glands in mutant rats reflect hyperplasia rather than cellular hypertrophy. Eph (erythropoietin-producing human hepatocellular receptors) signalling may be involved in the maintenance of adrenocortical zonation in rats [30]. Here, we detected a significant increase of Ephrin B1 gene expression in adrenal glands in GR^+/−^ rats on a standard diet (Figure 2F). In conclusion, we observed that GR haploinsufficiency in rats affects steroid and glucocorticoid synthesis and secretion and leads to adrenal hyperplasia.

### 2.2. GR^+/−^ Rats Developed Salt-Sensitive Hypertension

**Figure 3:**
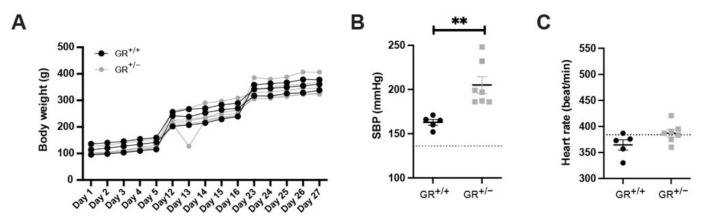
GR^+/−^ rats develop salt-sensitive hypertension. (**A**) Body weight, (**B**) systolic blood pressure (SBP) and (**C**) heart rate of GR^+/+^ (n = 4–5) and GR^+/−^ (n = 5–7) rats under high salt diet. The dotted lines (**B**,**C**) represent SBP and heart rate under standard diet. Values are indicated as mean ± SEM, and data were evaluated by two-way ANOVA and compared with Tukey test and differences assessed at ** *p* < 0.01.

We next challenged the animals with prolonged high salt diet. During the salt treatment, GR^+/+^ and GR^+/−^ rats ate and drank similarly, leading to an equal sodium intake (Figure S4A–C). GR^+/+^ and GR^+/−^ rats also showed similar urine output decreasing from the third week onwards (Figure S4D). The sodium and potassium excretion between GR^+/+^ and GR^+/−^ rats was similar (Figure S4E,F). Finally, the body weight of the GR^+/−^ was comparable to GR^+/+^ rats (Figure 3A). Following five weeks of high salt diet exposure, we observed a significant weight increase of the adrenal glands in GR^+/−^ compared to GR^+/+^ rats (Table S2). Blood pressure measurements revealed that both GR^+/+^ and GR^+/−^ rats had higher systolic blood pressure compared to the standard salt diet (dotted line) (Figure 3B). Furthermore, we observed that the GR^+/−^ rats had statistically higher systolic blood pressure compared to GR^+/+^ rats at high salt diet (Figure 3B), accompanied with a tendency of higher heart rate of the GR^+/−^ rats on high salt diet (Figure 3C). Only GR^+/−^ rats under high salt diet had increased kidney weight compared to GR^+/+^ rats (Table S2).

**Figure 4:**
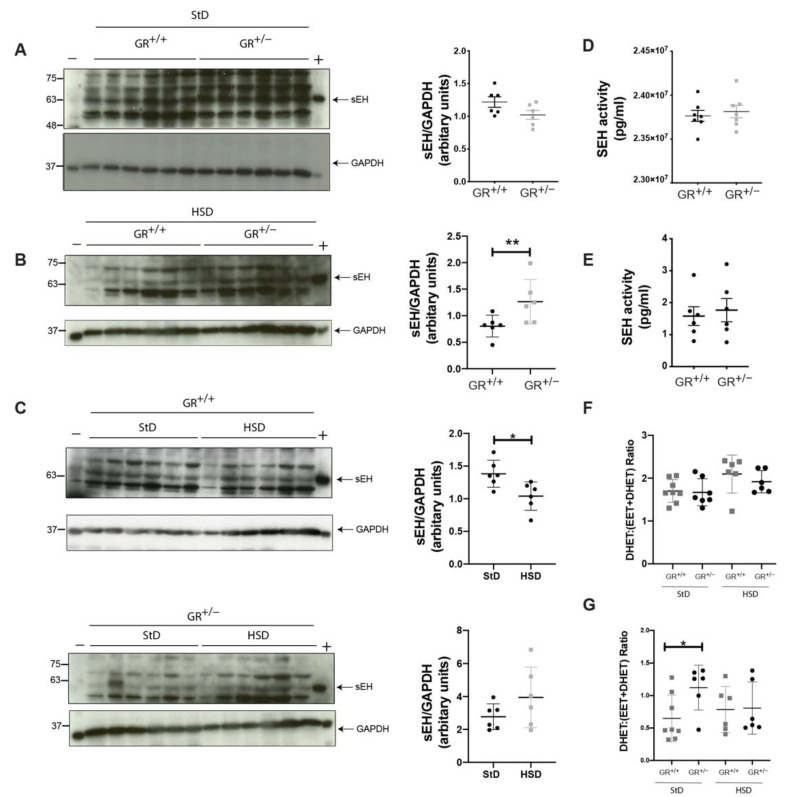
sEH protein levels remain elevated in GR^+/−^ rats under high salt diet. (**A**) Representative Western blot analysis and quantification of sEH protein expression under standard diet and (**B**) under high salt diet; (**C**) representative Western blot analysis and quantification of sEH protein expression in the adrenal glands of GR^+/+^ rats (n = 6) under standard and high salt diet (upper panel) and representative Western blot analysis and its quantification of sEH protein expression in the adrenal glands of GR^+/−^ rats (n = 6) under standard and high salt diet; (**D**) enzymatic activity of sEH in the adrenal glands of GR^+/+^ (n = 6) and GR^+/−^ rats (n = 6) under standard diet; (**E**) enzymatic activity of sEH in the adrenal glands of GR^+/+^ (n = 6) and GR^+/−^ rats (n = 6) under high salt diet; (**F**) sEH activity was estimated indirectly by the molar ratios of individual and total DHET/(EET + DHET) for arachidonic acids and (**G**) for linoleic acids. Values are indicated as mean ± SEM, and data were evaluated by unpaired two-tailed t test and differences assessed at * *p* < 0.05, ** *p* < 0.01.

It is known that glucocorticoids cause hypertension through several mechanisms [31], and here we focused on changes in vasodilation and/or vasoconstriction. Here, soluble epoxide hydrolase (sEH) plays an important role [32]. Therefore, we analysed the protein expression of sEH in the adrenal gland on standard diet (Figure 4A) and after five weeks of high salt diet (Figure 4B). On standard diet, GR^+/+^ and GR^+/−^ rats presented the same sEH protein level in the adrenal glands (Figure 4A). Nevertheless, we observed significant increased sEH protein levels in adrenals of GR^+/−^ compared to GR^+/+^ rats on high salt diet (Figure 4B). In addition, sEH protein abundance was reduced after high salt diet in GR^+/+^ rats (Figure 4C, upper panel), while those of GR^+/−^ rats remained elevated (Figure 4C, lower panel). Furthermore, we directly quantified the activity of the sEH in the adrenals. Under standard and high salt diet, this activity, per quantity of protein, is not significantly different in adrenal glands from GR^+/+^ and GR^+/−^ rats (Figure 4D,E). We also quantified indirectly the global sEH activity for the hydrolyzation of the arachidonic and the linoleic acids (∑DHET/(∑EET + ∑DHET) [33] (Figure 4F,G). The global sEH activity was similar in GR^+/+^ and GR^+/−^ rats for the arachidonic and the linoleic acids after high salt diet (Figure 4F,G). However, under standard diet, the global activity of sEH for the linoleic acid was significantly higher in GR^+/−^ rats (Figure 4G). Here, we identified that GR haploinsufficiency in rats leads to a salt-sensitive hypertension development accompanied by a lack of decrease of the adrenal sEH.

### 2.3. Salt-Sensitive Hypertension in GR^+/−^ Rats Is Associated with Changes within the Fatty Acids Metabolism

**Figure 5:**
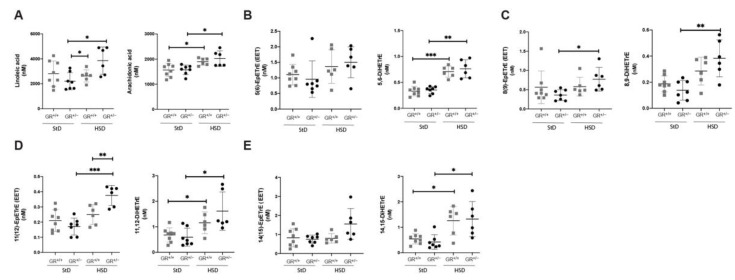
Fatty acid disturbances in arachidonic acid pathway of GR^+/−^ rats. Metabolites of (**A**) linoleic and arachidonic acid (**B**) 5(6)-EET and 5,6 -DiHETrE, (**C**) 8(9)-EET and 8,9 -DiHETrE, (**D**) 11(12)-EET and 11,12 -DiHETrE, (**E**) 14(15)-EET and 14,15 -DiHETrE measured in plasma of GR^+/+^ (StD n = 8, HSD n = 6) and GR^+/−^ rats (StD n = 7, HSD n = 6) under standard or high salt diet. Values are indicated as mean ± SEM, and data were evaluated by two-way ANOVA and compared with Tukey test, and differences assessed at * *p* < 0.05, ** *p* < 0.01 or *** *p* < 0.001.

To better understand the underlying mechanism, we analysed fatty acid metabolites (Figure S5) implicated in vasodilation and/or vasoconstriction. With respect to the arachidonic acid pathway (Figure 5), analysis of plasma revealed that the precursor of linoleic acid (Figure 5A) was higher in GR^+/−^ rats under high salt diet compared to GR^+/−^ rats on standard diet. Furthermore, we observed differences in DHET levels which are known to attenuate the action of EETs leading to a significant decrease of the vasodilation. Both GR^+/+^ and GR^+/−^ rats showed an increase of arachidonic acid, 5,6DiHETrE, 11,12- DiHETrE, and 14,15- DiHETrE following high salt diet, and 8,9- DiHETrE was additionally increased in GR^+/−^ rats (Figure 5B–E).

**Figure 6:**
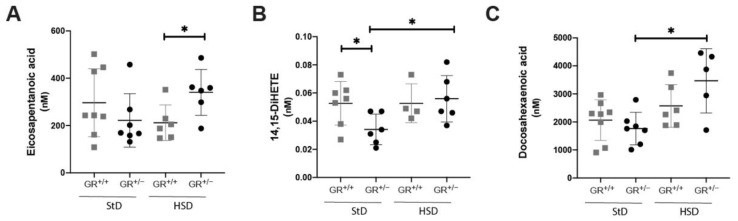
Fatty acid disturbances in eicosapentanoic and docosahexaenoic acid pathways of GR^+/−^ rats. Metabolites of (**A**) eicosapentanoic acid, (**B**) 14,15-DiHETrE and (**C**) docosahexaenoic acid measured in plasma of GR^+/+^ (StD n = 8, HSD n = 6) and GR^+/−^ rats (StD n = 7, HSD n = 6) under standard or high salt diet. Values are indicated as mean ± SEM, and data were evaluated by two-way ANOVA and compared with Tukey test and differences assessed at * *p* < 0.05.

In eicosapentanoic acid pathway (Figure 6A), we observed that eicosapentaenoic acid level was statistically higher in GR^+/−^ rats compared to GR^+/+^ rats on high salt diet. Furthermore, its downstream product 14,15-DiHETE (Figure 6B) was statistically lower in GR^+/−^ compared to GR^+/+^ rats under standard diet and was increased only under high salt diet reaching the same level as in GR^+/+^ rats. In GR^+/−^ rats, the docosahexaenoic acid level (Figure 6C) was significant increased following high salt diet compared to standard diet.

**Figure 7:**
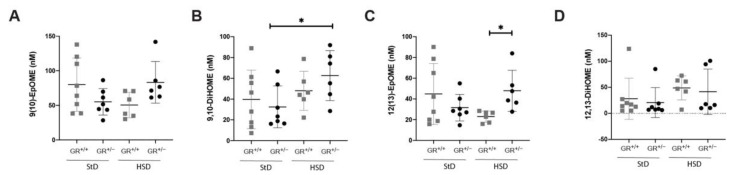
Fatty acid disturbances in linoleic acid pathway in GR^+/−^ rats. Metabolites of (**A**) 9,10-epoxyoctadecenoic acid (EpOME), (**B**) 9,10- dihydroxyoctadecenoic acid (DiHoME), (**C**) 12,13-EpOME and (**D**) 12,13-DiHoME measured in plasma of GR^+/+^ (StD n = 8, HSD n = 6) and GR^+/−^ rats (StD n = 7, HSD n = 6) under standard or high salt diet. Values are indicated as mean ± SEM, and data were evaluated by unpaired two-tailed *t* test and differences assessed at * *p* < 0.05.

In the linoleic acid pathway (Figure 7), no difference was found for the 9,10-epoxyoctadecenoic acid (EpOME) after the high salt diet (Figure 7A), but the corresponding diol 9,10- dihydroxyoctadecenoic acid (DiHoME) was significantly increased in GR^+/−^ rats (Figure 7B). Inversely, 12,13 EpOME level was significantly higher in GR^+/−^ rats on high salt compared to the GR^+/+^ rats (Figure 7C), whereas 12,13 DiHOME was expressed similarly in both groups (Figure 7D). This analysis revealed that, after five weeks of high salt diet, GR^+/−^ heterozygous rats developed salt-sensitive hypertension without downregulation of adrenal sEH, resulting in dysregulation of the arachidonic acid, the eicosapentanoic acid, and the linoleic acid pathways (Figure S5).

## 3. Discussion

GR mutations cause primary generalized glucocorticoid resistance (PGGR), also named Chrousos syndrome. PGGR is a condition characterized by generalized partial tissue insensitivity to glucocorticoids [34] that results in different clinical symptoms as adrenocortical hyperplasia and steroid disturbances [14,35]. Several observed human GR mutations in PGGR syndrome (76%) are also linked with the development of hypertension. However, despite this high rate of hypertension, the exact mechanism is unclear. Thus far, in order to study the role of GR in different pathologies, mouse and rat models carrying GR mutations have been used. Rats are the preferred animal model to study hypertension based on several criteria as the similarity to human disease, feasibility and size of the animals, and animal welfare considerations [36]. Therefore, we used the previously generated [29] GR^+/em2^ mutant rats to study the effect of GR haploinsufficiency on adrenal gland function and to reveal a possible pathway leading to hypertension in PGGR syndrome.

The GR^+/em2^ mutant rat is a heterozygous knockout rat model expressing 50% of wild-type GR protein, while the homozygous knockout rat (GR^−/−^) does not express any truncated GR and results in perinatal death of offspring (data not shown). This observation is in agreement with the previously described phenotype in GR^KO^ mice that die due to severe lung atelectasis [12,18,29].

In our study, we observed that GR^+/−^ rats have adrenal hyperplasia due to expansion of *zona fasciculata* (Figure 2), which also has an effect on steroid production, as evidenced by increased aldosterone and corticosterone levels, without an elevation of 11β-HSD2 activity (Figure 1). This phenotype is in line with the clinical symptoms described in four families with original heterozygous non-sense mutations showing a significant reduction of the GR transcript in fibroblasts: R469X [18] and R491X in the recent French MUTA-GR study [37] and E198X [38] and Y660X [39]. These patients presented adrenal hyperplasia, high cortisol but low aldosterone level. Patients are hypertensive and adrenal hyperplasia is only mentioned for E198X mutation. Overall, the GR^+/−^ mutant rats develop a primary generalized glucocorticoid resistance syndrome sharing several phenotypes with human. Elevated adrenal *ephb1* mRNA levels (Figure 2) might well correlate with the observed hyperplasia of *zona fasciculata* and is relevant for cellular orientation and migration within the adrenal gland zones in rodents [30,40,41,42,43] and humans [44].

The higher levels of corticosterone and aldosterone in combination with unchanged 11β-HSD2 activity suggested enhanced aldosterone-dependent MR activation and that the MR was probably not protected against higher levels of corticosterone, leading to a risk of hypertension development [45]. The GR^+/−^ rat model and the R491X patient share increased glucocorticoids, adrenal gland hyperplasia and a normotension at baseline (Figure 1 and Figure 2). Furthermore, serum aldosterone is low in the R491X patient but high in GR^+/−^ rats (Figure 1). Thus, a change in the steroid profile alone is not sufficient to develop hypertension. High salt diet ranging from 4–8% of salt was often used in Dahl salt-sensitive rats [46,47,48,49], and GR^+/+^ and GR^+/−^ rats ate and drank similarly during the high salt challenge (Figure S4A,B). Food and water intake declined after three weeks in both groups likely due to a salt retention, or the age of the rat [50,51]. Higher systolic blood pressure (Figure 3) and an increase of the left adrenal weight highly suggest that steroid disturbances in rodents are not sufficient to provoke hypertension and additionally salt is needed to induce hypertension.

Although similar levels of sEH protein abundances were found in GR^+/−^ and GR^+/+^ rats on standard diet, this protein decreased only in wild-type rats under high salt diet (Figure 4) indicating that it may confer a protective mechanism to limit an increase of blood pressure as it metabolises the vasodilatory EETs to less potent DHETEs [25,26]. It has been demonstrated that EETs are released from the zona glomerulosa cells through adrenocorticotropin hormone (ACTH) stimulation [52]. Besides, to the best of our knowledge, our study is the first reporting the presence of sEH in adrenal tissue at protein level. To note, sEH regulation was not extensively studied in high salt diet conditions and controversially discussed. In kidney, sEH was overexpressed in wild-type Wistar rats after 21 days feed with a 2% salt diet [53], whereas the enzyme was downregulated in eNOS^+/+^ and eNOS^−/−^ mouse aortas after five weeks fed with 4% salt diet [54].

sEH is furthermore implicated in the metabolism of the omega-3 and 6 polyunsaturated fatty acids, namely the arachidonic acid (AA), the eicosapentanoic acid (EPA), and the docosahexaenoic acid (DHA). The derived active fatty acids, epoxyeicosatrienoic acids (EETs), epoxyeicosatetraenoic acids (EEQs), and epoxydocosapentanoic acids (EDPs) [55], are hydrolysed by sEH to form the inactive dihydroxyeicosatrienoic acid (DHETs), dihydroxyeicosatetraenoic acid (DiHETEs), and dihydroxyeicosatetraenoic acid (DiHDPEs) (Figure S5) [55]. The role of the EETs in the regulation of the blood pressure has been demonstrated/confirmed in spontaneous hypertensive rats [27]. Besides, EETs were usually increased under high salt diet [56]. In Dahl salt-sensitive rats, an alteration in EETs secretion provoked hypertension [57]. Recently it was demonstrated that fatty acid oxidation in foetal cardiomyocytes is regulated by glucocorticoids [58].

Therefore, we quantified the metabolites of the arachidonic acid, the eicosapentanoic acid and the docosahexanoic acid in the plasma of GR^+/+^ and GR^+/−^ rats on standard and high salt diet. We observed that in GR^+/−^ rats, the absence of sEH downregulation in adrenal tissue after five weeks of high salt diet is associated with increase of EET (11(12)-EpETrE), eicosanoic acid, linolenic acid and its metabolite 12(13)-EpOME (Figure 5, Figure 6 and Figure 7). These data highly suggest that fatty acid metabolism may be implicated in salt-sensitive hypertension in GR^+/−^ rats (Figure 5).

The findings of the study showed that sEH and fatty acids may play a role in development of hypertension in PGGR. However, further experiments with administration of sEH inhibitors, similar to previous studies [59], will clarify whether sEH inhibition has possible protective effect against hypertension. These experiments will also unveil if sEH is implicated directly in blood pressure regulation or if it is a consequence of the overall changes in metabolism.

In conclusion, we demonstrated that GR haploinsufficiency in Sprague Dawley rats induced PGGR syndrome, leading to an adrenocortex hyperplasia, disturbances of the steroids profile and salt-sensitive hypertension that is associated with changes in fatty acids metabolites. This study linked, for the first time, the role of the adrenal soluble epoxide hydrolase (sEH) in PGGR to glucocorticoid resistance. These results suggest that sEH might be a new pharmaceutical target to treat hypertensive patients with GR haploinsufficiency. We focused in our work on the adrenal gland and the consequences of changed sEH levels and fatty acids metabolites. It will be interesting to further analyse the endothelial function in such rat models.

## 4. Materials and Methods

### 4.1. Rats

GR mutant rats (GR^+/em2^, referred also as GR^+/−^) were generated [29] by mating heterozygous GR mutant and wildtype rats of a Sprague Dawley genetic background. Offspring were genotyped for the GR gene locus at 5-8 days of age by PCR and RNA sequence. Animal maintenance and experimental procedures were in agreement with the Swiss federal guidelines and were approved by veterinarian local authorities of the Canton de Vaud, Switzerland (#VD3333, 14.06.2018). The animals were housed in a temperature- and humidity-controlled room with an automatic 12 h light/dark cycle (light: 7 a.m. to 7 p.m.). The rats were housed in ventilated cages at 23 ± 1°C with free access to food (StD 0.25% Na^+^ and 0.70% K^+^ diet, Provimi Kliba AG, Switzerland) and tap water. In our experiments, we used 4–5 weeks old male rats. The rats were kept on standard diet (StD,0.25% Na^+^ w/w) or on high salt diet (HSD, 0.39% Na^+^ in water and 2.2% Na^+^ in food) for 5 weeks [46,47,60]. Over this period, blood pressure was measured always at the same time of the day (14:00–16:00 a.m.).

### 4.2. Metabolic Cages Studies

Four- to five-week-old rats were individually placed into metabolic cages for 6 days: one day for the adaptation and 5 days of measurements. During the experiment, the body weight, food and water consumption, amount of urine and faeces were determined daily. During the experiment, the rats were subjected to different salt diets as indicated for each experiment. Urine and faeces were collected for measuring the composition of the electrolytes [Na^+^, K^+^]. Between two metabolic cage sessions, rats were placed in a normal stock cage for 10 days.

### 4.3. PCR

Rats were genotyped for the disrupted *Nr3c1* gene locus by PCR using genomic DNA biopsies and a set of three primers; GR1a (intron 2-Forward) and GR2b (intron 3-Reversed) (see primers list) (Table S1, Figure S2). DNA was stored at −20 °C until analysis. PCR reaction for *Nr3c1* contained 2 × buffer S, 0.2 mM dNTPs, 0.5 mM of each primer (GR1a and GR2b), and 2.5 U Taq polymerase (peqGOLD Taq DNA Polymerase). The PCR program for *Nr3c1* was constituting of 36 cycles; each run consisted of 1 min at 95 °C, followed by 1 min at 60 °C and 1 min at 72 °C. PCR reactions generated a 453 base pair fragment for wild type *Nr3c1* or a 419 base pair fragment for mutant *Nr3c1*, depending on GR genotype. In order to distinguish the wild type from mutant band, the PCR product was digested with BstNI (Biolabs) according to the instructions. Only the mutant product can be digested by BstNI and thus generated two fragments (274 bp and 172 bp), which were analysed on 3% agarose gels (Figure 1A). RT-PCR of rat GR transcripts was performed with total RNA isolated from fibroblasts of embryos using the Takara PrimeScript RT reagent kit. Primers were from the rat GR gene; GR1c (exon 2), GR1d (exon 3), GR1e (exon 4), GR3a (exon 8), GR3b (exon 8) (see primers list) (Figure S2). RT-PCR was performed with 10 mM primers, as described by Takara (PrimeScript RT reagent Kit). PCR reaction contained 1 × Buffer S, 0.2 mM dNTPs, 0.5 mM of each primer, and 2.5 U Taq polymerase (peqGOLD Taq DNA Polymerase). The PCR analysis for *Nr3c1* was performed in 35 cycles; each run consisted of 30 s at 94 °C, followed by 30 s at 72 °C and 1 min at 72 °C. For exons 2–3 and exon 8 amplification the reaction was performed at 59.9 °C and for exons 2–4 at 58.7 °C. Products were analysed on 3% agarose gels.

### 4.4. Isolation of Rat Embryonic Fibroblasts (REFs)

Primary REF cells were isolated from embryos of wild-type (WT) and GR^em2^ homozygous rats at embryonic day 13.5 as described [61]. Briefly, embryos were removed and separated from maternal tissues and yolk sac and were finely minced, digested with 0.05% trypsin/1 mM EDTA for 30 min at 37 °C, and centrifuged for 5 min at 1000× *g*. The pellet was filtered and resuspended in culture medium before plating. Cells were cultured at 37 °C in high glucose DMEM (Invitrogen) supplemented with 10% (*v*/*v*) FBS (Omega Scientific) and 100 units/mL penicillin/streptomycin (Invitrogen). REF cells were frozen in liquid nitrogen at passage 1 in aliquots of 1 × 10^6^ cells/vial. In the experiments REFs were used no later than passage 4. All treatments of cells were carried out in charcoal dextran stripped serum-containing DMEM.

### 4.5. Western Blot Analysis

Liver or kidney extracted proteins (25 μg homogenate) were resolved on 10% polyacrylamide gels by SDS-PAGE electrophoresis, transferred to a nitrocellulose membrane, and then incubated with a GR-specific antibody ((rabbit anti-GR (N-terminal, M-20; Santa Cruz, CA, USA), rabbit anti-GR (C-terminal amino-acids 755–771, PA1-516, Thermo Fischer Scientific)). sEH expression was analysed in 30 µg of protein lysate (rabbit anti-sEH, Cayman Chemicals, 10010146). Immunoreactive bands were visualized by chemiluminescence (ECL kit; Amersham Biosciences, 152 Little Chalfont, UK). An anti-actin monoclonal (rabbit anti-actin (A2066, Merck)) or a mouse anti-GAPDH antibody (Merck) were used to verify equal protein loading between samples.

### 4.6. Histology

Paraffin blocks of adrenal gland samples were used to prepare serial sections for haematoxylin and eosin staining and immunohistochemical analysis. Slides were immunostained with monoclonal rabbit antibody selective to rat CYP11B2 (kind gifts of Celso Gomez-Sanchez) on a Ventana BenchMark ULTRA system following the manufacturer’s instructions.

### 4.7. Steroid Profile Analysis

Blood was collected through cardiac puncture in wild type or mutant rats at 7–8 a.m. or 6–7 p.m. after anaesthesia with 1 to 2% isoflurane. Plasma was isolated with centrifuge at 5000–6000 rpm for 20 min at 4 °C. Steroid hormones were quantified by ultra-performance liquid chromatography-tandem mass spectrometry (LC-MSMS) as described earlier, with minor adaptations using an Agilent 1290 Infinity II UPLC coupled to an Agilent 6495 MS/MS instrument [62].

### 4.8. Blood Pressure

Intra-arterial blood pressure (BP) was recorded by PE-10 catheter with a computerized data acquisition system in conscious rats using previously described method with an adaption [63], in brief; rats were anesthetized via inhalation of 1 to 2% isoflurane mixed with oxygen. The right carotid artery was exposed for a length of approximately 4 mm. A PE-10 catheter filled with 0.9% NaCl solution containing heparin (300 IU/mL) was inserted into the artery. After ligation, the catheter was subcutaneously tunnelled to exit at the back of the neck. The rat was allowed 3 h to recover from the anaesthesia and was placed in a Plexiglas tube for partial restriction of their movements. Thirty minutes later, the arterial line was connected to a pressure transducer and blood pressure was measured 2–4 p.m.

BP and heart rate (HR) were then monitored every 20s for 15 to 30 min by Notocord HEM 3.1 software (SA, Croissy, France) at a sampling rate of 500 Hz. After the BP measurements, the rats were sacrificed, and the heart and kidneys were excised and weighed. Cardiac and renal hypertrophy was determined by heart and kidney indices (heart or kidney weight/body weight, mg/g).

### 4.9. Echography

Following a protocol previously described [64], the animals were anesthetized for 10 min with isoflurane 4% + oxygen (1 L/min) and placed on a heating table (37−38 °C) to keep the body temperature constant. During the procedure, vital parameters such as cardiac frequency, body temperature, and breath rate were monitored. The transducer collected the sounds that bounced back, and a computer then used those sound waves to create an image (Visualsonics, Vevo 2100 model).

### 4.10. Statistics

Values are indicated as mean ± SEM, and data were evaluated by unpaired two-tailed *t* test in Figure 2, Figure 4, Figure 7 and Figure S4, and differences assessed at * *p* < 0.05, ** *p* < 0.01 or *** *p* < 0.001. Values are indicated as mean ± SEM, and data were evaluated by two-way ANOVA and compared with Fisher’s LSD test in Figure 1A–D, and differences assessed at * *p* < 0.05; ** *p* < 0.01; *** *p* < 0.001. Values are indicated as mean ± SEM, and data were evaluated by two-way ANOVA and compared with Tukey test in Figure 3, Figure 5 and Figure 6, and differences assessed at * *p* < 0.05, ** *p* < 0.01 or *** *p* < 0.001. Values are indicated as mean ± SEM, and data were evaluated by two-way ANOVA and compared with Fisher’s LSD test in Tables S2 and S3, and differences between standard diet and high salt diet assessed at *p < 0.05; **p < 0.01; ***p < 0.001 and differences between GR^+/+^ and GR^+/−^ rats assessed at ^#^ *p* < 0.05; ^##^ *p* < 0.01; ^###^ *p* < 0.001

## 5. Conclusions

GR hyploinsufficiency in Sprague Dawley rats induced PGGR syndrome similar to humans with adrenal gland hyperplasia, hormone disturbances and salt-sensitive hypertension. Furthermore, we here detected a metabolic switch of fatty acid oxidation accompanied with changes in adrenal sEH levels. These observations suggested that sEH might be a promising pharmaceutical target to treat hypertensive patients with GR haploinsufficiency.

## Supplementary Materials

The following are available online at https://www.mdpi.com/article/10.3390/ijms222413218/s1.
