# Supplementary material for: Salt-Sensitive Hypertension in GR+/− Rats Is Accompanied with Dysregulation in Adrenal Soluble Epoxide Hydrolase and Polyunsaturated Fatty Acid Pathways
Source: Int J Mol Sci. 2021 Dec 8;22(24):13218. doi: 10.3390/ijms222413218 (PMC8708190; doi:10.3390/ijms222413218)
Supplement: Supplementary file 1 [file ijms-22-13218-s001.zip › ijms-1469510-supplementary.pdf]

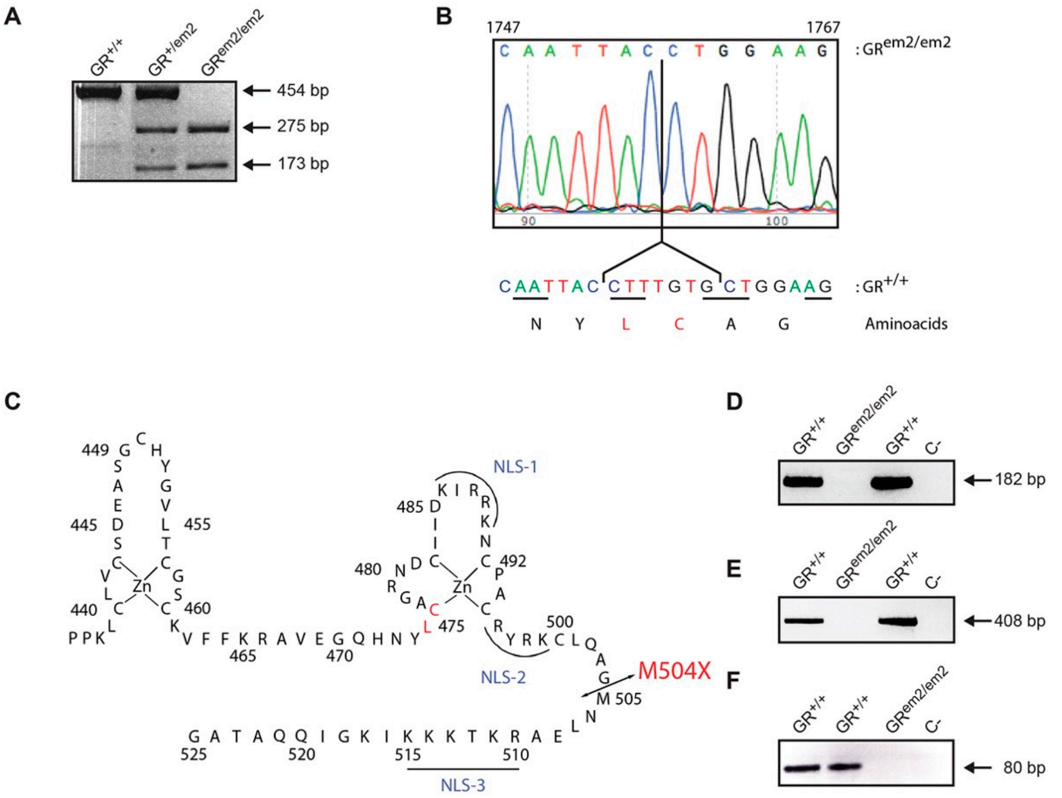

Figure S1. GR em2 mutation is an out of frame deletion**.** (A) Genotyping of wild-type ($GR^{+/+}$), heterozygous ($GR^{+/em2}$) and homozygous ($GR^{em2/em2}$) mutant rats using restriction enzyme digestion (BstNI) (B) Sequence analysis of the exon 3 of *Nr3c1* in wild-type ($GR^{+/+}$) and homozygous ($GR^{em2/em2}$) mutant rats (C) Schematic representation of the rat GR DBD. The DBD consists of two zinc fingers and includes three nuclear localization signals (NLS 1-3). The deleted amino acids and the early stop codon appearing in position 504 are indicated in red. (D) Detection of *Nr3C1* transcripts (exon 2 (forward:1c) and exon 3 (reversed:1d)) in kidney of wild-type and homozygous mutant rats. E) Detection of *Nr3C1* transcripts (exon 2 (forward:1c) and exon 4 (reversed:1e)) in kidney of wild-type and homozygous mutant rats. F) Detection of *Nr3c1* mRNA transcripts (exon 8 (forward:3a) and exon 8 (reversed:3b)) in kidney of wild-type and homozygous mutant rats.

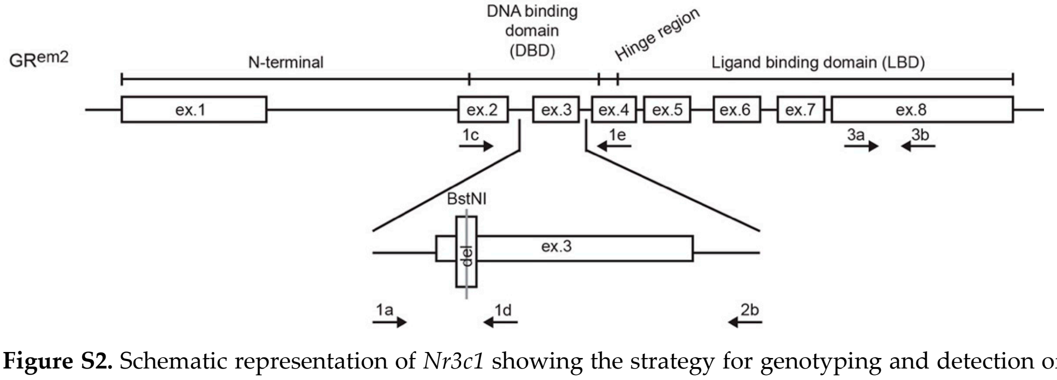

Figure S2. Schematic representation of *Nr3c1* showing the strategy for genotyping and detection of transcripts GR^{em2}. Positions of primers within the different exons are marked with arrows. For genotyping forward 1a and reversed 2b were used and the amplicon further digested by BstNI enzyme. While for transcript detection forward 1c and reversed 1d (182bp wt amplicon) or forward 1c and reversed 1e (182bp wt amplicon) or forward 3a and reversed 3b (80bp wt amplicon) were used.

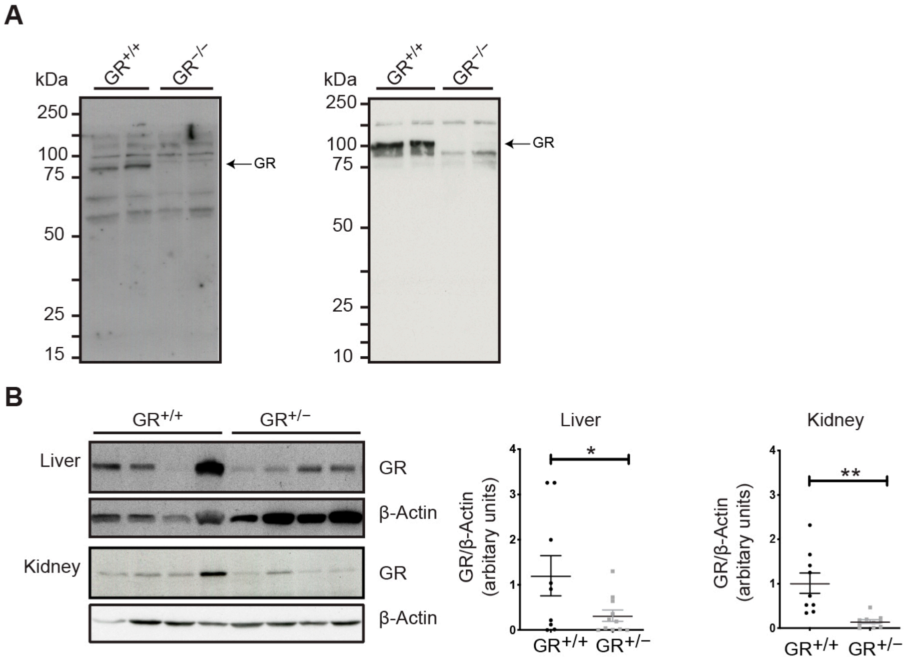

Figure S3. GR^{em2} mutant protein is not translated (A) Protein extracts from rat embryonic fibroblasts were analyzed by Western blot analysis using a polyclonal GR antibody directed to the N-terminus (left panel) or a polyclonal GR antibody directed to the C-terminus (right panel). (B) Representative Western blot analysis and its quantification of GR and actin abundance in whole liver (upper panel) or kidney (lower panel) lysates of 3-weeks old GR$^{+/+}$ (n=9) and GR$^{-/-}$ (n=10) using a polyclonal N-terminal GR antibody. Differences between GR$^{+/+}$ and GR$^{+/-}$ rats were analyzed by t-test and significant differences assessed at *P<0.05 or **P<0.01.

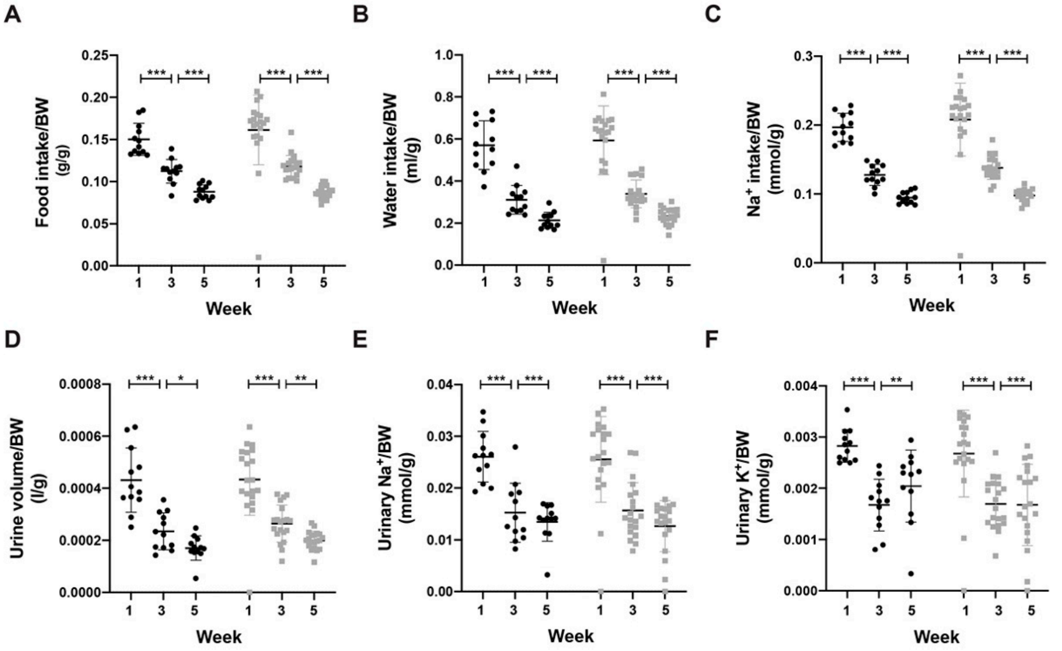

Figure S4. Metabolic cage studies in GR$^{+/+}$ and GR$^{+/-}$ feeding 1, 3 and 5 weeks of high salt diet. (A) food intake per weight, (B) water intake per weight, (C) sodium intake per weight, (D) urine volume per weight, (E) urinary sodium per weight and (F) urinary potassium per weight in GR$^{+/+}$ (n=3) and GR$^{+/-}$ (n=5) rats in high salt diet. Values are indicated as mean±SEM, and data were evaluated by two-way ANOVA and compared with Tukey test in and differences assessed at *P <0.05, **P <0.01 or ***P <0.001.

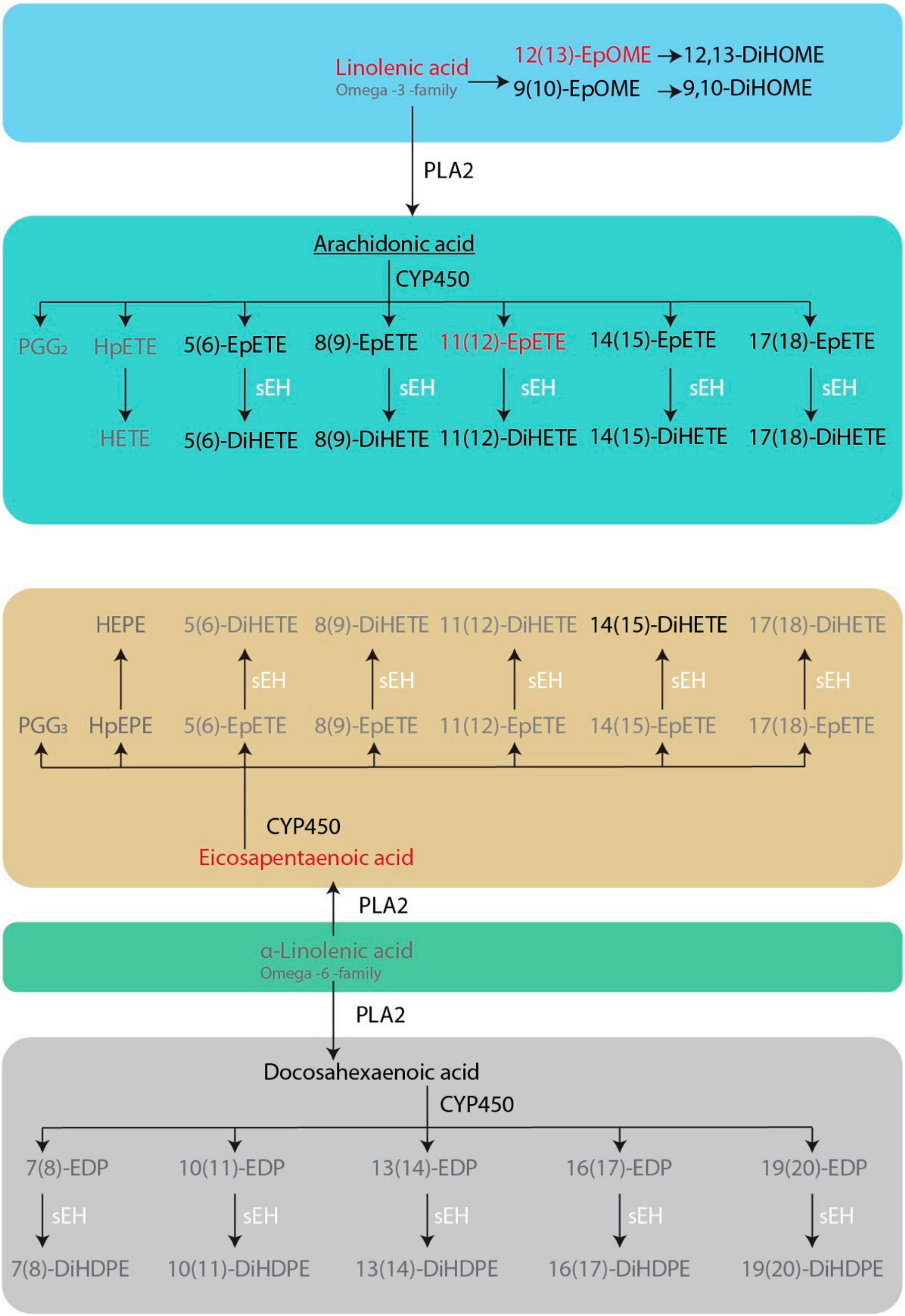

Figure S5. Fatty acids pathway**.** Grey are the fatty acids that weren't measured and red are the fatty acids that upregulated in $GR^{+/-}$ compared with $GR^{+/+}$ in high salt diet.

Table S1 Primer list. Primer list

Gene	Primer	Sequence
Nr3C1	1a (F)	5'-CTC TCA ACA TGG TAA TTC ATG TAG AAA AG-3'
	1b (R)	5'-GCT GCT CAG ACT CAG GCA C-3'
	1c (F)	5'-TAA GCT CTC CTC CAT CCA GCT-3'
	1d (R)	5'-GCA ATC GTT TCT TCC AGC ACA-3'
	1e (R)	5'-TCA ATC ACC TCC AGC AGT GAC -3'
	2a(F)	5'-GTC TGT GGA ATT TTA ACA ATG C-3'
	2b(R)	5'-GGA CAG CCA GGG TGT ATA TT-3'
	3a(F)	5'-AGC TGT TTA AGA TGG GCA GCT-3'
	3b(R)	5'-GAA GCA CCG ACC CAT TTT CAC-3'
Ephb1	F	5'-TGC AGC AGG AAA CGA GCT TA-3'
	R	5'-ATC TCC TTG GCA AAC TCC CG-3'
GAPDH	F	5'-CAT GGC CTT CCG TGT TCC TA-3'
	R	5'-CCT GCT TCA CCA CCT TCT TGA-3'

Table S2 Organs weight of $GR^{+/+}$ and $GR^{+/-}$ rats under standard salt (StD) and high salt diet (HSD). Heart weight (HW), kidney weight (KW) or adrenal gland weight (AdrW) corrected by the body weight (BW). Values are indicated as mean±SEM, and data were evaluated by two-way ANOVA and compared with Fisher's LSD test and differences between standard diet and high salt diet assessed at *P<0.05; **P<0.01; ***P<0.001 and differences between $GR^{+/+}$ and $GR^{+/-}$ assessed at $^{\#}$P<0.05; $^{\#\#}$P<0.01; $^{\#\#\#}$P<0.001

Parameters	Diet	$GR^{+/+}$	n	$GR^{+/-}$	n
**HW/BW (mg/g)**	StD	2.9±0.41	4	3.3±0.17	5
	HSD	4.7±0.77**	6	5.4±0.42***	7
**KW/BW (mg/g)**	StD	7.9± 0.51	4	8.3±0.91	5
	HSD	12.0±0.69***	6	16.0±2.92$^{***,\#\#}$	7
**AdrW/BW (mg/g)**	StD	0.12±0.01	2	0.2±0.06	2
	HSD	0.16±0.02*	6	0.3±0.09$^{\#\#}$	7

Table S3 Plasma steroid profile– of GR$^{+/+}$ and GR$^{+/-}$ rats . Table of steroids measured in plasma of GR$^{+/+}$ and GR$^{+/-}$ rats in the morning (A.M.) and in the afternoon (P.M.). Values are indicated as mean±SEM, and data were evaluated by two-way ANOVA and compared with Fisher's LSD test, differences between GR$^{+/+}$ and GR$^{+/-}$ assessed at *P<0.05; **P<0.01; ***P<0.001, and differences between morning (A.M.) and afternoon (P.M.) assessed at #P<0.05, ##P<0.01.

Hormone	Time	GR$^{+/+}$	n	GR$^{+/-}$	n
11-dehydrocorticosterone (nM)	A.M.	50.28±5.97	15	76.63±5.25***	16
	P.M.	74.61±5.63 ##	8	101.80±4.02**,##	8
17a-Hydroxyprogesterone (nM)	A.M.	0.21±0.05	8	0.13±0.03	8
	P.M.	0.09±0.01	8	0.18±0.03	8
Testosterone (nM)	A.M.	0.57±0.09	15	0.47±0.08	16
	P.M.	0.60±0.02	8	0.74±0.09 #	8
Androstendiol (nM)	A.M.	6.73±0.95	8	11.1±1.26*	8
	P.M.	9.81±0.99	8	13.75±1.70*	8
Androstenedione (nM)	A.M.	0.51±0.10	15	0.26±0.0.06*	16
	P.M.	0.28±0.10	8	0.21±0.04	8
Androsterone (nM)	A.M.	6.75±2.14	13	1.96±0.56*	11
	P.M.	1.88±0.28#	8	1.90±0.29	8
Androstendiol / Testosterone	A.M.	9.32±1.28	8	17.42±2.86**	8
	P.M.	16.73±1.46##	8	19.00±0.98	8
